# Intersectoral costs of sexually transmitted infections (STIs) and HIV: a systematic review of cost-of-illness (COI) studies

**DOI:** 10.1186/s12913-021-07147-z

**Published:** 2021-10-29

**Authors:** Lena Schnitzler, Louise J. Jackson, Aggie T. G. Paulus, Tracy E. Roberts, Silvia M. A. A. Evers

**Affiliations:** 1 Health Economics Unit, Institute of Applied Health Research, College of Medical and Dental Sciences, University of Birmingham, Birmingham, UK; 2 Department of Health Services Research, Care and Public Health Research Institute (CAPHRI), Faculty of Health, Medicine and Life Sciences (FHML), Maastricht University, Maastricht, The Netherlands; 3 School of Health Professions Education (SHE), Faculty of Health, Medicine and Life Sciences (FHML), Maastricht University, Maastricht, The Netherlands; 4 Trimbos Institute, Centre for Economic Evaluations, Netherlands Institute of Mental Health and Addiction, Utrecht, The Netherlands

**Keywords:** Sexually transmitted infections, STIs, HIV, Cost-of-illness, Intersectoral costs, Economic burden of disease

## Abstract

**Background:**

Sexually transmitted infections (STIs) and HIV can generate costs both within and outside the health sector (i.e. intersectoral costs). This systematic review aims (i) to explore the intersectoral costs associated with STIs and HIV considered in cost-of-illness (COI) studies, (ii) to categorise and analyse these costs according to cost sectors, and (iii) to illustrate the impact of intersectoral costs on the total cost burden.

**Methods:**

Medline (PubMed), EMBASE (Ovid), Web of Science, CINAHL, PsycINFO, EconLit and NHS EED were searched between 2009 and 2019. Key search terms included terms for cost-of-illness, cost analysis and all terms for STIs including specific infections. Studies were included that assessed intersectoral costs. A standardised data extraction form was adopted. A cost component table was established based on pre-defined sector-specific classification schemes. Cost results for intersectoral costs were recorded. The quality of studies was assessed using a modified version of the CHEC-list.

**Results:**

75 COI studies were considered for title/abstract screening. Only six studies were available in full-text and eligible for data extraction and narrative synthesis. Intersectoral costs were captured in the following sectors: *Patient & family, Informal care* and *Productivity (Paid Labour).* Patient & family costs were addressed in four studies, including patient out-of-pocket payments/co-payments and travel costs. Informal care costs including unpaid (home) care support by family/friends and other caregiver costs were considered in three studies. All six studies estimated productivity costs for paid labour including costs in terms of absenteeism, disability, cease-to-work, presenteeism and premature death. Intersectoral costs largely contributed to the total economic cost burden of STIs and HIV. The quality assessment revealed methodological differences.

**Conclusions:**

It is evident that intersectoral costs associated with STIs and HIV are substantial. If relevant intersectoral costs are not included in cost analyses the total cost burden of STIs and HIV to society is severely underestimated. Therefore, intersectoral costs need to be addressed in order to ensure the total economic burden of STIs and HIV on society is assessed, and communicated to policy/decision-makers.

**Supplementary Information:**

The online version contains supplementary material available at 10.1186/s12913-021-07147-z.

## Background

Sexually transmitted infections (STIs) remain a health threat to millions of people [1]. Healthcare costs for STIs and human immunodeficiency virus (HIV), particularly direct medical costs (i.e., drugs, hospitalisation), represent a substantial cost burden on society [2, 3]. However, STIs and HIV can also have an impact on the wider economy, affecting other sectors of society such as labour, households and education [4, 5]. Costs associated with a disease that occur both within and outside the health sector are typically defined as *societal* [6], *multisectoral* [7] or *intersectoral* costs [8].

Cost-of-illness (COI) studies are a commonly used framework designed to identify, measure and value the costs incurred by society due to a particular disease [9, 10]. The consideration of intersectoral costs in these studies can generate useful information fundamental for optimal policy/decision-making, including the process of resource allocation to optimise population health and to justify the necessity of an intervention [9, 10].

The majority of existing COI studies, however, primarily consider healthcare costs and, as a consequence, potentially underestimate the total cost burden of a disease to society [11]. An underestimation of the complete cost burden could lead to an inefficient use and distribution of public health resources. A more comprehensive picture of the costs associated with STIs and HIV is crucial to increasing the prioritisation of STIs and HIV on the public health agenda and in the wider political arena, and is important in making the case for more financial support for the area of sexual health.

A societal perspective is often considered appropriate for COI studies, as it allows us to capture all relevant costs in economic analyses [12], but not all studies adopt such a perspective. In some countries where national health economic guidelines require taking a societal perspective, such as in the Netherlands [13], it is vital to consider all relevant costs associated with a disease including healthcare costs and costs spilling over to other sectors (i.e. intersectoral costs). Other countries including the United Kingdom often adopt a healthcare (or National Health Service (NHS)) perspective and predominantly assess costs falling on the healthcare sector [14], but there is acknowledgment of the benefit of considering a wider perspective in the analysis [15]. The increasing interest by national authorities in capturing intersectoral costs of public health problems in economic studies reflects the importance of these costs [15]. It is notable that the consideration of the intersectoral impacts of a disease has also received more prominence in light of the current COVID-19 pandemic and could potentially shape the way in which economic assessments are done moving forward [16, 17].

Studies exist that identify, measure and value intersectoral costs in areas such as mental health [8] and alcohol prevention programmes [18]﻿, for example, but are limited in the field of sexual health. To date, the wider intersectoral impacts of STIs and HIV on society are relatively unexplored but could be significant given the rising STI rates and growing demand for sexual health services [19]. The current review aims (i) to explore the intersectoral costs associated with STIs and HIV considered in COI studies, (ii) to categorise and analyse these costs according to cost sectors, and (iii) to illustrate the impact these intersectoral costs can have on the total cost burden of STIs and HIV.

## Methods

Prior to conducting the systematic review, a protocol was registered and published with PROSPERO (Registration Number: CRD42019130940) [20].

### Search strategy

An extensive search strategy was developed in PubMed as part of a larger systematic literature review including COI and economic evaluation studies (Additional file 1). Relevant key search terms for this present review included terms for cost-of-illness, cost analysis and all terms for STIs including specific infections. Seven databases were searched: Medline (PubMed), EMBASE (Ovid), Web of Science, CINAHL, PsycINFO, EconLit and NHS EED, limiting studies to 2009-2019. Reference lists of selected articles were screened.

### Inclusion criteria

Studies were included that assessed costs beyond healthcare costs and were conducted in an Organisation for Economic Co-operation and Development (OECD) member country. The country scope was chosen for better evaluation of comparable health systems and policies. Studies were selected that focused on STIs that were sexually transmitted, and included participants of at least 12 years or older.

### Selection of papers

Search results were exported into EndNote X9. Citations were systematically de-duplicated following the guidelines by Bramer and colleagues [21]. The study selection was performed by two reviewers (LS, LJ). A three-stage process was adopted to guide the screening of studies for inclusion [22]. In stage I, one independent reviewer (LS) screened articles on the basis of titles only, followed by stage II title and abstract screening. The same reviewer categorised included studies into groups as either a (A) cost-of-illness study or (B) economic evaluation. Studies were further categorised by disease; (a) Chlamydia, (b) Gonorrhoea, (c) Trichomoniasis, (d) Herpes/HSV, (e) HIV, (f) HPV, (g) Syphilis, (h) Hepatitis B, and (i) more than one STI. Studies other than a COI study or economic evaluation were excluded. A second reviewer (LJ) reviewed this process and discrepancies were discussed. This review’s analysis focused on A) COI studies of all diseases (a-i). COI studies were screened for full-text in Stage III. A standardised data extraction form was adopted and modified for the purpose of this review [8, 23].

### Analysis

Data were recorded in Microsoft Excel and Word and analysed narratively. A cost component table was established with inspiration from pre-defined sector-specific classification schemes to inform the analysis [8, 24]. Cost results for intersectoral costs were listed, categorised and analysed. While assessing the impact of intersectoral costs on the total costs, the reported costs were converted to US Dollars and the year 2021, adjusting the values by inflation. This was done using an online inflation tool [25] and a currency converter [26].

### Quality assessment

No single standard quality assessment tool exists for COI studies. The quality of studies was assessed using a modified version of the Consensus on Health Economic Criteria (CHEC) list (Additional file 2) [27]. The guide for critical evaluation of COI studies by Larg & Moss (2011) was also considered [28]. The quality assessment was not used to mediate articles for inclusion/exclusion, but to inform the analysis. The results for the quality assessment are presented narratively.

## Results

**Fig. 1:**
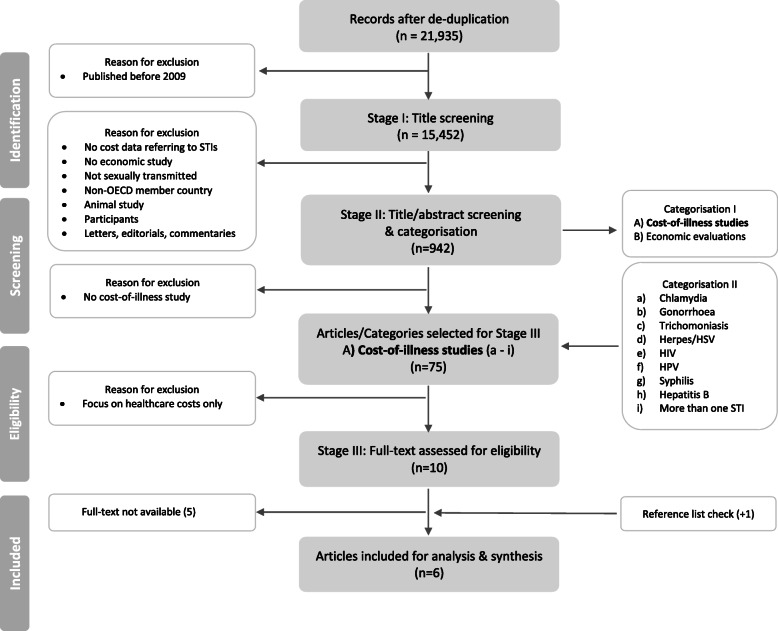
PRISMA Flowchart

The search strategy, as part of a larger systematic literature review, identified 21,935 articles after de-duplication. Due to the high number of records identified between 1999 and 2019, studies were further limited to 2009-2019, which led to the exclusion of 6,426 studies. This time period was selected to reflect the greater attention focused on incorporating intersectoral costs in economic analyses over the past ten years [15, 29, 30]. Studies were excluded that did not mention any cost data concerning STIs. Seventy-five COI studies were considered for title/abstract screening. Studies were further excluded that assessed healthcare costs only. Ten studies were eligible for full-text screening of which five were only available in form of an abstract or poster (Additional file 3). Corresponding authors were contacted. One study was found by screening the reference lists of the other five studies selected for analysis [31]. Ultimately, six studies were available for full-text analysis and qualified for data extraction and narrative synthesis, having considered intersectoral costs in their analyses, see Fig. 1.

### Study characteristics

Table 1 presents a summary of the study characteristics. Included studies were conducted in Germany, Spain, the United States and South Korea. The societal approach is the recommended approach for ensuring the total cost of a disease is captured, and was fully adopted by four studies [5, 31–33]. The other two studies used a combination of a societal and payer perspective; both were carried out in Germany [34, 35]. A prevalence-based approach is appropriate when assessing the total costs of a disease within a specific timeframe and was followed by three studies [31–33]. The remaining studies did not explicitly report the epidemiological approach taken. A bottom-up costing approach can record the quantity of resource use at an individual level ensuring all relevant costs are captured, and was followed by the three studies that all focused on HIV [32, 34, 35]. The remaining three studies used evidence from claims data or other aggregated data [5, 31, 33], which is typical for a top-down approach. Two studies reported to have used a prospective study design [34, 35], whereas one used a retrospective approach [32].

**Table 1:** Study characteristics

Authors	Year	Country	Type of STI	Perspective	Epidemiological approach	Resource quantification	Study design	Time horizon	Year of valuation	Currency
Kuhlmann et al.	2015	Germany	HIV	Payer (SHI), Societal	NR	Bottom-up	Pro	2009-2011	2009	EUR
Lopez-Bastida ﻿et al.﻿	2009	Spain	HIV	Societal	P	Bottom-up	Re	2013	2003	EUR
Mostardt et al.	2013	Germany	HIV	Payer (SHI), Societal	NR	Bottom-up	Pro	2006-2009	2008	EUR
Owusu-Edusei et al.	2013	USA	Non-viral STIs (Chlamydia, Gonorrhoea, Syphilis, Trichomoniasis)	NR (Societal)	NR	NR	NR	2001-2005	2011	USD
Shon et al.	2015	South Korea	Hepatitis A B C	Societal	P	NR	NR	2008-2011	Average exchange rate during 2008-2011	USD
Yang et al.	2010	South Korea	Hepatitis B	Societal	P	NR	NR	2005	2005	KRW

*EUR *Euro, *HIV* Human Immunodeficiency Virus, *KRW* Korean Won, *NR *Not reported, *P *Prevalence, *Pro *Prospective, *Re *Retrospective, *SHI *Statutory Health Insurance, *USD *United States Dollar

### Intersectoral cost components

Table 2 shows which intersectoral costs are included in the selected studies. Five of the six studies estimated healthcare costs and costs in at least one of the following sectors: *Patient & family, Informal care* and *Productivity (Paid labour)* [31–35]. One study assessed productivity (labour) costs only [5].

**Table 2:** Intersectoral costs identified in the selected studies

Cost components per sector/ Authors		Kuhlmann et al.	Lopez-Bastida et al.	Mostardt et al.	Owusu-Edusei et al.	Shon et al.	Yang et al.	Total
**PATIENT & FAMILY**		**√**		**﻿√**		**﻿√**	**﻿√**	**4**
Out-of-pocket costs as part of health systems/insurance co-pay ^a^		﻿√				﻿√	﻿√	
Out-of-pocket costs outside health systems/insurance ^b^							﻿√	
Out-of-pocket costs for hired caregiver							﻿√	
Travel expenses for patients				﻿√		﻿√	﻿√	
Travel expenses for family/caregiver							﻿√	
**INFORMAL CARE**				**﻿√**		**﻿√**	**﻿√**	**3**
Time invested/productivity lost by non-paid family/friends				﻿√			﻿√	
Caregiver support for outpatient care						﻿√*		
**PATIENT PRODUCTIVITY - PAID LABOUR**		**﻿√**	**﻿√**	**﻿√**	**﻿√**	**﻿√**	**﻿√**	**6**
Productivity loss due to absenteeism ^c^		﻿√		﻿√	﻿√	﻿√	﻿√	
Productivity loss due to morbidity			﻿√				﻿√	
Productivity loss due to disability				﻿√				
short-term				﻿√				
long-term				﻿√				
partial				﻿√				
Productivity loss stemming from cease-to-work							﻿√	
Productivity loss due to premature death						﻿√	﻿√	

^a^ Categorised and assessed alongside healthcare costs (direct costs) in the original study. It includes patient out-of-pocket co-payments for medical services and drugs not covered by the national health insurance. Kuhlmann et al. referred to it as patient costs (or *Patientenkosten* and *Patientenzuzahlungen)*.

^b^ Categorised and assessed alongside healthcare costs (informal direct medical costs) in the original study. It includes over-the-counter drugs, dietary supplements, folk remedies, traditional Korean medicine services and other treatment-related resource utilizations paid for by patients.

^c^ Three studies (Owusu-Edusei et al., Shon et al., and Yang et al.) accounted for patient time lost, for instance, for care-seeking. The studies equated these to productivity or income lost.

* It was not clear whether caregivers involved paid or unpaid support and whom this involved (i.e. friends, family).

Patient & family costs were addressed in four studies, with some costs being related to healthcare services and treatment within a national insurance system and others to patient-borne expenses outside an insurance system [31, 33–35]. Kuhlmann et al. estimated patient out-of-pocket payments or co-payments for their antiretroviral treatment that were not fully covered by their health insurer [34]. Healthcare costs that were not covered by the insurer and co-payments for medical services and drugs were also captured by Shon et al. and Yang et al. [31, 33]. Other treatment-related costs paid for by the patient included over-the-counter drugs, dietary supplements, folk remedies and other traditional Korean medicine services [31]. Yang et al. also captured patient out-of-pocket costs for hired caregivers [31]. Expenditures incurred by the patient for traveling to medical visits were estimated in three studies [31, 33, 35]. In addition to patient transportation costs, Yang et al. also captured the travel costs incurred by caregivers [31]. All three studies classified travel expenses incurred by the patient or caregiver under healthcare costs (direct non-medical costs).

Two studies explicitly reported to have captured informal care costs, which were concerned with unpaid (home) care support by family or friends [31, 35]. Mostardt et al. captured home care provided by family/friends as part of direct costs (healthcare costs) [35], whereas Yang et al. classified and calculated time and productivity costs by caregivers as part of indirect costs (non-health costs) [31]. A third study, Shon et al., also captured caregiver costs as part of indirect costs (non-health costs) but did not specify whether caregivers involved paid or unpaid support and whom this involved (i.e. family, friends) [33].

Productivity costs can involve productivity losses for paid labour and non-paid opportunity costs (i.e. leisure time, domestic work). All six studies estimated patients’ productivity costs for paid labour, including costs in terms of absenteeism, short-term/partial/long-term disability, cease-to-work, presenteeism or premature death [5, 31–35]. One study assessed productivity (labour) losses for outpatient patients only [5].

### The impact of intersectoral costs on the total costs of STIs and HIV

The impact of HIV-related productivity costs per year per patient on the overall costs were presented in three studies and varied between 9 % and 41 % [32, 34, 35], see Table 3. For non-viral STIs, the average productivity loss per case was estimated at $262 for chlamydia, $197 for gonorrhoea, $419 for syphilis and $289 for trichomoniasis [5]. The study did not estimate the healthcare costs involved, therefore, it was not possible to illustrate the additional impact productivity costs have on the total costs. However, the authors argued that productivity losses related to non-viral STIs might be higher than healthcare costs. For hepatitis A, B and C, opportunity costs lost (as a result of seeking medical care, or premature death and caregiver costs) represented 65 %, 53.4 % and 42.0 % of the total costs, respectively [33]. The intersectoral costs for Hepatitis B as estimated by Yang et al. represented around 75.5 % of the total costs [31].

**Table 3:** The impact of intersectoral costs on the total costs of STIs and HIV

Authors	Healthcare costs	Patient/family costs	Productivity (labour) costs	Total intersectoral costs	Total costs (healthcare and intersectoral)	Proportion of intersectoral costs on the total cost (%)
Kuhlmann et al. (HIV)	Total healthcare costs: 22,457 €/year [2009-2011] per patient	Patient out-of-pocket costs: 216 €/year per patient	1,890 €/year per patient	2,106 €/year per patient	24,563 €/year per patient	9 %
	[US$ 2021: 29.437]	[US$ 2021: 283]	[US$ 2021: 2,474]	[US$ 2021: 2,765]	[US$ 2021:32.200]	
Lopez-Bastida et al. (HIV)	Total healthcare costs (asymptomatic HIV): 7,148 €/year [2003] per patient	NA	Asymptomatic HIV: 3,383 €/year per patient	3,383 €/year per patient	Asymptomatic HIV: 10,531 €/year per patient	“Productivity losses for people living with HIV to range between 3,383€ (asymptomatic HIV) and 5,981€ (symptomatic HIV), representing a range of 32-41 % of the total costs.” [as reported in the original study]
	[US$ 2021: 10,611]		[US$ 2021: 5,023]	﻿[US$ 2021: 5,023]	[US$ 2021: 15,635]	
	Total healthcare costs (symptomatic HIV): 8,508 €/year per patient		Symptomatic HIV: 5,981 €/year per patient	5,981 €/year per patient	Symptomatic HIV: 14,489 €/year per patient	
	[US$ 2021: 12,632]		[US$ 2021: 8,879]	﻿[US$ 2021: 8,879]	[US$ 2021: 21,512]	
Mostardt et al. (HIV)	Total healthcare costs (SHI): 19,103 €/year [2008] per patient*	NA	Disability-related productivity loss (labour): 489 €/year per patient	489 €/year per patient	23,298 €/year per patient	“9 % of total cost from the societal perspective could be attributed to indirect costs [disability, productivity loss].” [as reported in the original study]
	[US$ 2021: 25,865]		[US$ 2021: 662]	﻿[US$ 2021: 662]	[US$ 2021: 31,542]	
			Long-term productivity loss (labour): 1,294 €/year per patient	1,294 €/year per patient		
			[US$ 2021: 1,752]	[US$ 2021: 1,752]		
			Partial productivity loss (labour): 337 €/year per patient	337 €/year per patient		
			[US$ 2021: 456]	[US$ 2021: 456]		
Owusu-Edusei et al. (non-viral)	NA	NA	Average productivity costs (labour) per case/2001-2005 [2011 values]: US$ 262 for chlamydia [US$ 2021: 312]	US$ 262 for chlamydia per case	NA	NA
			US$ 197 for gonorrhoea [US$ 2021: 234]	US$ 197 for gonorrhoea per case		
			US$ 419 for syphilis [US$ 2021: 498]	US$ 419 for syphilis per case		
			US$ 289 for trichomoniasis [US$ 2021: 344]	US$ 289 for trichomoniasis per case		
Shon et al. (Hepatitis A, B, C)	NA	NA	NA	NA	Hepatitis A: US$ 45.7 million/2008-2011 [US$ 2021: 54,3 million]	“[…] with indirect costs [opportunity costs lost as a result of medical care, or premature death and caregiver costs] accounting for the remaining 65 % during the observation period [2008-2011].” (Hepatitis A)
					Hepatitis B: US$ 607.8 million/2008-2011 [US$ 2021: 722.8 million]	“Indirect costs were estimated to be approximately 53.4 % of this total over the same period [2008-2011]” (Hepatitis B)
					Hepatitis C: US$ 90.7 million/2008-2011 [US$ 2021: 107.8 million]	“[…] with indirect costs accounting for the remaining 42.0 % in 2011.” (Hepatitis C) [as reported in the original study]
Yang et al. (Hepatitis B)	Direct costs (direct formal medical costs, informal medical costs, and non-medical costs): 474,642 million KRW/year [2005] [or 0.474,642 trillion]**	NA [refer to the column on the right]	Indirect costs (time costs, caregiver costs, productivity losses): 1.463 trillion KRW/year incurred by HBV-related disease patients	1.463 trillion KRW/year incurred by HBV-related disease patients	1.937 trillion KRW/year	75,5 %
	[US$ 2021: 558,639,140]		[US$ 2021: 1,721,832,880]	[US$ 2021: 1,721,832,880]	[US$ 2021: 2,279,692,610]	

*KRW* Korean Won, *NA *Not applicable, *SHI *Statutory health insurance, *US *United States Dollar

*In the original study, patient travel costs and costs for homecare provided by family/friends were included in the calculation of healthcare costs.

**In the original study, patient costs (patient resource consumption outside the health care system: dietary supplements, over the counter drugs, other treatment-related services) and transportation costs were included in the calculation of direct costs (healthcare costs).

The reported costs were converted to US Dollar and the year 2021, adjusting the values by inflation.

### Quality assessment

The quality of the COI studies did not vary considerably but methodological differences were evident (Additional file 2). Five studies mentioned the study perspective adopted for analysis [32–35]. The type of epidemiological approach taken was explicitly reported by three studies, in this case a prevalence-based approach [31–33]. Three studies explicitly reported they had used a bottom-up approach for resource use quantification [32, 34, 35]. The same studies also stated the type of study design, with two assessing data prospectively [34, 35] and one retrospectively [32]. One study did not disclose any information regarding the choice of methodological approaches such as perspective, resource quantification, study design or epidemiological approach [5]. The time horizon for assessment, year of cost evaluation and currency were reported in all studies. Important costs were identified, measured and valued in five studies in relation to the perspective and the study objectives [31–35]. One study limited their analysis to productivity (labour) costs, though, this was also in line with their research objectives [5]. None of the studies discounted future costs. Sensitivity analysis was conducted in one study [31]. Almost all studies discussed the generalisability of results.

## Discussion

### Principal findings

This review is the first to explore whether existing COI studies carried out for STIs and HIV considered intersectoral costs in their analyses, and to categorise these according to specific cost sectors. Further, it clearly demonstrates that intersectoral costs significantly contribute to the total cost burden of STIs and HIV.

Only a small number of COI studies were identified that captured intersectoral costs of STIs and HIV. This small number implies that intersectoral costs are often overlooked in the literature and remain largely excluded from COI studies in this area. Some might argue this finding could indicate that intersectoral costs are not relevant, but this review concludes differently. For example, this review provides evidence that productivity losses for people living with HIV can account for up to 40 % of the total costs per year.

There are several reasons that could explain why many studies ignored the wider scope of costs, even though intersectoral costs for STIs and HIV can be substantial [5]. One reason could be a more narrow study perspective applied, for instance, to inform decision-makers in the health sector that might only be interested in the costs paid from the health budget. Another reason could be feasibility in terms of the lack of time, resources or data available for the wider analysis. Yet another reason for a narrow focus on costs might be the lack of realisation of the importance of intersectoral costs, particularly in COI studies. As mentioned earlier such lack of realisation might have changed in light of the current COVID-19 pandemic that has exposed the larger intersectoral impacts of health issues on society [17, 36]. The importance of considering these wider costs is evident and fundamental in order to avoid the risk of omitting important costs to inform decision-making, in both health and other sectors.

This review suggests that the COI studies that adopted a societal perspective tend to predominantly assess healthcare and productivity costs related to paid labour. This is in line with previous research reporting that even when a societal perspective is adopted in economic studies these often only consider healthcare and labour costs [37]. The focus on healthcare and productivity costs could be explained by the fact that traditional approaches to COI studies broadly divide costs into direct, indirect and intangible costs [38]. The included studies reveal that the biggest impact of intersectoral costs was in the labour market. It was suggested that productivity losses could potentially be greater than healthcare costs [5].

The present findings suggest that the assessment of unpaid labour and non-paid opportunity costs such as leisure time, volunteering and care for children or elderly is rather limited in COI studies. Similar findings were found for full economic evaluations (i.e. cost-effectiveness analysis) [39], however, the inclusion of these types of economic evaluations associated with interventions for STIs is explored elsewhere [PROSPERO, Reference ID: CRD42019130940].

As mentioned earlier, intersectoral costs can have a big impact on the total cost burden. In fact, this review reveals that the inclusion of intersectoral costs attributed to STIs and HIV indicate a substantially higher cost burden to society than healthcare costs alone. This means that unless intersectoral costs are taken into account, the total cost implications of STIs will not be appreciated. This is in keeping with a review of COI studies by Pike and colleagues (2015) which reported that limiting the assessment of the economic burden to healthcare costs can substantially underestimate the total economic cost burden [40].

This review found that the heterogeneity of methodological approaches in COI studies, including the choice of study perspective(s) and what costs to include in analysis, made it rather difficult to analyse and compare the impact of intersectoral costs across studies. The use of different methods in cost analyses can affect how results are interpreted and subsequently affect policy decisions. This review raises awareness on the potential need for standardised guidelines for COI studies and a standard quality assessment tool for COI studies to assess the consistency and transparency of these studies and improve comparability.

### Policy implications

The present review shows that only a small number of COI studies of STIs and HIV include intersectoral costs. Those studies that do capture intersectoral costs tend to report a higher burden for STIs and HIV, which is important information for policy/decision-makers. These findings imply that if intersectoral costs are not included in cost analyses, the total cost burden of STIs and HIV to society is severely underestimated. If intersectoral costs are captured in COI studies this may change the overall results and is likely to improve the information developed for decision/policy-makers. Realising the higher cost burden of STIs and HIV might give more prioritisation to interventions targeted at reducing the number of STIs and HIV compared with other competing demands on the healthcare budget.

### Strengths and weaknesses of the study

The strength of this review lies in its rigorous and systematic approach. A comprehensive search strategy was developed in collaboration with an information specialist. Studies were carefully screened to evaluate whether intersectoral costs had been captured and a cost component table (or classification scheme) was established that can be adapted or expanded by future research, as needed. The present review considered studies conducted in all OECD member countries to account for a good representation of study results. Further, this review was able to synthesise evidence that addresses the impact intersectoral costs of STIs and HIV can have on the total economic burden. This review also has some limitations. One limitation is that other studies that potentially assessed intersectoral costs may have been missed. The articles were limited to the timeframe of 2009-2019. Five potentially relevant articles eligible for full-text analysis were only available in form of abstracts or posters. After finding that only six studies were eligible for data extraction and narrative synthesis and available in full the review team scanned a random number of excluded studies to check whether potentially relevant articles may have been missed. Further, the complex nature of STIs and HIV requires an examination of the wider societal impacts, and the included studies might not represent the fuller range of potentially relevant cost sectors. This however also means that the present classification scheme could serve as a guide for future research and offers room for expansion.

Overall, this review has generated pertinent evidence and presents a clear message that the focus of most of the existing COI studies to date is largely on healthcare costs when it is evident that the impact of disease is wider and more substantial.

### Further research

Future research could further investigate relevant cost sectors associated with STIs and HIV and validate or complement the findings of this review. Gathering more evidence could help propose a standardisation of cost classifications for COI studies concerned with STIs and HIV. Economic evaluations could be reviewed to identify the different sector-specific costs as well as benefits associated with interventions targeting STIs and HIV.

## Conclusions

It is evident that intersectoral costs associated with STIs and HIV are substantial and largely contribute to the total economic cost burden. However, studies tend to predominantly assess healthcare and productivity costs related to paid labour under a societal perspective. If relevant intersectoral costs are not included in cost analyses the total cost burden of STIs and HIV to society is severely underestimated. Therefore, intersectoral costs associated with STIs and HIV need to be addressed in order to ensure the total economic burden of STIs and HIV on society is assessed, and communicated to policy/decision-makers.

## Supplementary information

**Additional file 1**

**Additional file 2**

**Additional file 3**

## Data Availability

The datasets used and/or analysed during the current study are available from the corresponding author on reasonable request.

## Abbreviations

CHEC: Consensus Health Economic Criteria
COI: Cost-of-illness
EUR: Euro
HIV: Human immunodeficiency virus
HPV: Human papillomavirus
HSV: Herpes simplex virus
KRW: Korean Won
OECD: Organisation for Economic Co-operation and Development
PRISMA: Preferred Reporting Items for Systematic Reviews and Meta-Analyses
PROSPERO: The International Prospective Register of Systematic Reviews
SHI: Statutory Health Insurance
STI: Sexually transmitted infection
USD: United States Dollar
