# Supplementary material for: Intersectoral costs of sexually transmitted infections (STIs) and HIV: a systematic review of cost-of-illness (COI) studies
Source: BMC Health Serv Res. 2021 Oct 29;21:1179. doi: 10.1186/s12913-021-07147-z (PMC8555721; doi:10.1186/s12913-021-07147-z)
Supplement: Supplementary file 1 — Additional file 1 [file 12913_2021_7147_MOESM1_ESM.docx]

Additional file 2: PubMed search strategy

((((((((("Sexual Behavior"[MeSH] OR sexual behavior*[tiab] OR sexual behaviour*[tiab] OR sexual activit*[tiab] OR sexual education[tiab] OR sex education[tiab] OR sexuality education[tiab] OR sexual health[tiab] OR "Sexual Health"[MeSH] OR safe sex[tiab] OR unsafe sex[tiab] OR "Unsafe Sex"[MeSH] OR "Contraception"[MeSH] OR contracepti*[tiab] OR birth control[tiab]) OR ("Sexually Transmitted Diseases"[MeSH] OR sexually transmitted disease*[tiab] OR sexually transmitted infection*[tiab] OR STD[tiab] OR STDs[tiab] OR STI[tiab] OR STIs[tiab] OR venereal disease*[tiab] OR "Acquired Immunodeficiency Syndrome"[MeSH] OR acquired immune deficiency syndrome*[tiab] OR Acquired Immunodeficiency Syndrome*[tiab] OR acquired immuno deficiency syndrome*[tiab] OR Acquired Immunologic deficiency Syndrome*[tiab] OR AIDS[tiab] OR herpes genitalis[tiab] OR genital herpes[tiab] OR "Syphilis"[MeSH] OR syphilis[tiab] OR "Chlamydia"[MeSH] OR "Chlamydia infections"[MeSH] OR chlamydia[tiab] OR "HIV"[MeSH] OR HIV[tiab] OR human immunodeficiency virus*[tiab] OR "Gonorrhea"[MeSH] OR gonorrh*[tiab] OR "Trichomonas Infections"[MeSH] OR trichomonas infection*[tiab] OR trichomonias*[tiab] OR "Pelvic Inflammatory Disease"[MeSH] OR pelvic inflammatory disease*[tiab] OR inflammatory pelvic disease*[tiab] OR HPV[tiab] OR human papillomavirus*[tiab] OR condylomata acuminata[tiab] OR genital wart*[tiab] OR venereal wart*[tiab] OR "Hepatitis B"[MeSH] OR "Hepatitis B"[tiab])) AND ((cost[tiab] OR costs[tiab] OR "costs and cost analysis"[MeSH:noexp] OR (cost benefit analyses[Tiab] OR cost benefit analys*[Tiab]) OR "cost-benefit analysis"[MeSH] OR "health care costs"[MeSH:noexp]) OR (("Cost of Illness"[MeSH] OR (health expenditure[tiab] OR health expenditure*[tiab]))))))))
