# Supplementary material for: Intersectoral costs of sexually transmitted infections (STIs) and HIV: a systematic review of cost-of-illness (COI) studies
Source: BMC Health Serv Res. 2021 Oct 29;21:1179. doi: 10.1186/s12913-021-07147-z (PMC8555721; doi:10.1186/s12913-021-07147-z)
Supplement: Supplementary file 2 — Additional file 2 [file 12913_2021_7147_MOESM2_ESM.docx]

Additional file 3: Quality assessment of cost-of-illness studies

| Criteria/First author | Kuhlman | Lopez-Bastida | Mostardt | Owusu-Edusei | Shon | Yang |
| --- | --- | --- | --- | --- | --- | --- |
| 1. Is the study population clearly described? | Y | Y | Y | - | Y | Y |
| 2. Is a well-defined research question posed in answerable form? | Y | Y | Y | Y | Y | Y |
| 3. Is the economic study design appropriate to the stated objective? | Y | Y | Y | Y | Y | Y |
| 4. Is the actual perspective chosen appropriate? | Y | Y | Y | Y | Y | Y |
| 5. Are all important and relevant costs identified? | Y | Y | Y | - | Y | Y |
| 6. Are all costs measured appropriately? | Y | Y | Y | Y | Y | Y |
| 7. Are costs valued appropriately? | Y | Y | Y | Y | Y | Y |
| 8. Are all future costs discounted appropriately? | - | - | - | - | - | - |
| 9. Are all important variables, whose values are uncertain, appropriately subjected to sensitivity analysis? | - | - | - | - | - | Y |
| 10. Do the conclusions follow from the data reported? | Y | Y | Y | Y | Y | Y |
| 11. Does the study discuss the generalisability of the results to other settings and patient/client groups? | Y | Y | Y | Y | - | Y |
| 12. Does the article indicate that there is (no) potential conflict of interest of study researcher(s) and funder(s)? | Y | Y | Y | Y | Y | * |
| 13. Does the study discuss important limitations regarding the cost components, data, assumptions and methods? (adopted from Larg & Moss, 2011) | Y | Y | Y | Y | Y | Y |
| Y=Yes  * The study, however, indicated under ‘Acknowledgments’ that it was supported in part by a research grant from a pharmaceutical corporation. | | | | | | |

In the study, results were presented narratively which means no scores were calculated.

The checklist is based on the following material:

- Criteria 1-12: Evers S, Goossens M, de Vet H, van Tulder M, Ament A. Criteria list for assessment of methodological quality of economic evaluations: Consensus on Health Economic Criteria. Int J Technol Assess Health Care. 2005;21(2):240-245.
- Criteria 13: Larg, A., & Moss, J. R. (2011). Cost-of-illness studies. *Pharmacoeconomics*, *29*(8), 653-671.
