# Supplementary material for: Intersectoral costs of sexually transmitted infections (STIs) and HIV: a systematic review of cost-of-illness (COI) studies
Source: BMC Health Serv Res. 2021 Oct 29;21:1179. doi: 10.1186/s12913-021-07147-z (PMC8555721; doi:10.1186/s12913-021-07147-z)
Supplement: Supplementary file 3 — Additional file 3 [file 12913_2021_7147_MOESM3_ESM.docx]

Additional file 4: List of the studies not available in full-text at the time of data analysis

E. C. Wolf, S. 2016. Health-related costs in chronic HIV infection: A case-control study versus general population using a claims-based approach in Germany

http://www.natap.org/2016/GLASGOW/2016posterComCostGermany_P153.pdf

M. K. Tatar, G.; Ozelgun, B.; Elbir, T. Z.; Senturk, A.; Tuna, E.; Unal, S.; Tumer, A.; Inkaya, C. 2016. Indirect Cost of Hiv/Aids: Results of a Survey from a Turkish Research Center

http://polarsaglik.com/uploads/polar/Indirect2.pdf

M. K. Stoll, A.; Hower, M.; Heiken, H.; Gerschmann, S.; Klauke, S.; Lutz, T.; Bogner, J.; Degen, O.; Van Lunzen, J.; Bachmann, C.; Stellbrink, H.; Schmidt, W.; Leistner, I.; Mahlich, J. C.; Ranneberg, B. 2012. Corsar-study (cost and resource utilisation study in antiretroviral treated patients)

https://www.sciencedirect.com/science/article/pii/S1098301512028136?via%3Dihub

L. L. Smylie, P.; Lerch, R.; Kennedy, C.; Bennett, R.; Clarke, B.; Diener, A. 2011. The economic burden of chlamydia and gonorrhoea in Canada

https://sti.bmj.com/content/87/Suppl_1/A156.1

R. W. B. Baran, R.; Kleinman, N.; Beren, I.; Dietz, B. 2012. Employees living with human immunodeficiency virus: Impact of disease and antiretroviral therapies on health care costs and productivity

https://betterhealthworldwide.com/wp-content/uploads/2015/05/Comparative-HIV-AMCP20121.pdf
